# Zerumbone Protects against Carbon Tetrachloride (CCl_4_)-Induced Acute Liver Injury in Mice via Inhibiting Oxidative Stress and the Inflammatory Response: Involving the TLR4/NF-κB/COX-2 Pathway

**DOI:** 10.3390/molecules24101964

**Published:** 2019-05-22

**Authors:** Meilin Wang, Jingling Niu, Lina Ou, Bo Deng, Yingyi Wang, Sanqiang Li

**Affiliations:** Medical College, Henan University of Science and Technology, Luoyang 471023, China; meilin11954@163.com (M.W.); 18438591806@163.com (J.N.); lina.ou@163.com (L.O.); deng_bo888@163.com (B.D.); yingyiwang2019@163.com (Y.W.)

**Keywords:** Zerumbone, acute liver injury, antioxidant, anti-inflammation, pro-inflammatory cytokine, TNF-α, IL-6, TLR4, NF-κB, COX-2

## Abstract

The natural compound Zerumbone (hereinafter referred to as ZER), a monocyclic sesquiterpenoid, has been reported to possess many pharmacological properties, including antioxidant and anti-inflammatory properties. This study aimed to investigate the underlying mechanism of ZER against acute liver injury (ALI) in CCl_4_-induced mice models. ICR mice were pretreated intraperitoneally with ZER for five days, then received a CCl_4_ injection two hours after the last ZER administration and were sacrificed 24 h later. Examination of serum aspartate aminotransferase (AST) and alanine aminotransferase (ALT) activities and the histopathological analysis confirmed the hepatoprotective effect of ZER. Biochemical assays revealed that ZER pretreatment recovered the activities of antioxidant enzymes superoxide dismutase (SOD) and glutathione peroxidase (GSH-Px), restored the glutathione (GSH) reservoir, and reduced the production of malondialdehyde (MDA), all in a dose-dependent manner. Furthermore, administration of ZER in vivo reduced the release amounts of pro-inflammatory cytokines interleukin-6 (IL-6) and tumor necrosis factor alpha (TNF-α) and inhibited the increased protein levels of Toll-like receptor 4 (TLR4), nuclear factor-kappaB (NF-κB) p-p65, and cyclooxygenase (COX-2). Further studies in lipopolysaccharide (LPS)-induced Raw264.7 inflammatory cellular models verified that ZER could inhibit inflammation via inactivating the TLR4/NF-κB/COX-2 pathway. Thus, our study indicated that ZER exhibited a hepatoprotective effect against ALI through its antioxidant and anti-inflammatory activities and the possible mechanism might be mediated by the TLR4/NF-κB/COX-2 pathway. Collectively, our studies indicate ZER could be a potential candidate for chemical liver injury treatment.

## 1. Introduction

The liver, the largest digestive gland in human body, not only carries out the metabolism of carbohydrates, proteins, and lipids, but is also involved in secretion and biotransformation. Meanwhile, as the main executer of detoxification, the liver is highly vulnerable to all kinds of xenobiotics, such as exogenous chemicals, viruses, many drugs, and bacteria metabolites [1]. Using drug-induced liver injury as an example, the annual incidence in the general population was estimated to be 23.80 per 100,000 persons in mainland China [2]. Clearly, hepatic disease has become a major global burden on human health. However, it is still a therapeutic challenge to develop hepatoprotective drugs with high efficacy and low side effects [1,3].

A large amount of evidence indicates that oxidative stress and inflammation are two of the most important pathogenic mechanisms for hepatic disease [4]. CCl_4_ is a well-known hepatotoxin. Acute administration of CCl_4_ to rodents, which rapidly leads to oxidative stress and an inflammatory response, is the most widely accepted in vivo experimental model [5,6] and is extensively used to identify new, effective drugs and investigate the underlying mechanisms [7,8]. The toxic effects observed in CCl_4_ intoxication come from a toxic metabolite of CCl_4_, trichloromethylperoxy radical, which triggers lipid peroxidation and oxidative stress [4,9]. CCl_4_ intoxication also causes localized necrosis of liver tissues, followed by the activation of Kupffer cells [10]. Together with the damaged hepatocytes, the activated Kupffer cells release chemokines and inflammatory cytokines, such as TNF-α and IL-6, which lead to an inflammatory response [7,11,12]. Healthy inflammation helps to clear the necrotic cell debris and stimulate regeneration of hepatocytes, however, unregulated inflammatory response can provoke further hepatic damage [13]. Many pharmaceutical interventions that are effective against acute liver injury (ALI) have shown the benefits of inflammation inhibition [14,15].

Toll-like receptors (TLRs) are a well-known family of pattern-recognition receptors, which are expressed in a series of cell types including Kupffer cells and other inflammatory cells [16,17]. During acute liver injury (ALI), necrosis of the hepatocytes gives rise to the release of cellular constituents such as ATP, mitochondrial DNA, and molecules like high mobility group box-1 protein (HMGB1), which are recognized as damage-associated molecular patterns (DAMPs) to trigger the TLRs [18]. TLR triggering leads to the production of pro-inflammatory cytokines and mediators such as TNF-α, IL-6, and COX-2 via the phosphorylation and activation of NF-κB [18,19,20].

Recently, various natural products extracted from traditional Chinese medicine plants have drawn great attention in treatment of liver diseases [7,12]. Zerumbone (ZER) is a monocyclic sesquiterpenoid isolated from *Zingiber zerumbet* Smith, a perennial medicine herb. It has many biomedical properties, such as antioxidant, anti-inflammatory, anti-allergic, and anti-neoplastic properties [21]. Over the past few years, evidence has accumulated showing that ZER is a multi-targeted phytochemical with potential to treat many diseases, including osteoarthritis [22], diabetic complications [23,24], atherosclerosis [25], neuropathic pain [26], acute lung injury [27], and cancer, through the modulation of various molecular targets and cellular signaling cascades [21,28].

ZER is reported to have pharmacological activity against hepatic disease, including nonalcoholic fatty liver disease, chronic liver fibrosis, and ALI [29,30,31]. However, the underlying mechanism for ALI has not yet been well-studied. Therefore, the present study was undertaken to explore the hepatoprotective mechanism of ZER against ALI in vivo and in vitro. A CCl_4_-induced ALI mice model was employed. ICR mice were pretreated intraperitoneally with ZER at 1.25, 5, and 20 μmol/kg for five days, then received a CCl_4_ injection two hours after the last ZER administration. Then, the oxidative stress levels and the extent of the inflammatory response was evaluated. Our in vivo data indicate that ZER exhibited hepatoprotective effects against ALI by alleviating oxidative stress and inhibiting the inflammatory response and the possible mechanism might be mediated by the TLR4/NF-κB/COX-2 pathway. Our in vitro experiments confirmed that ZER could inhibit the inflammatory response through the TLR4/NF-κB/COX-2 pathway.

## 2. Results

### 2.1. Effects of ZER on Serum Aspartate Aminotransferase (AST), Alanine Aminotransferase (ALT), and Liver Index

**Figure 1:**
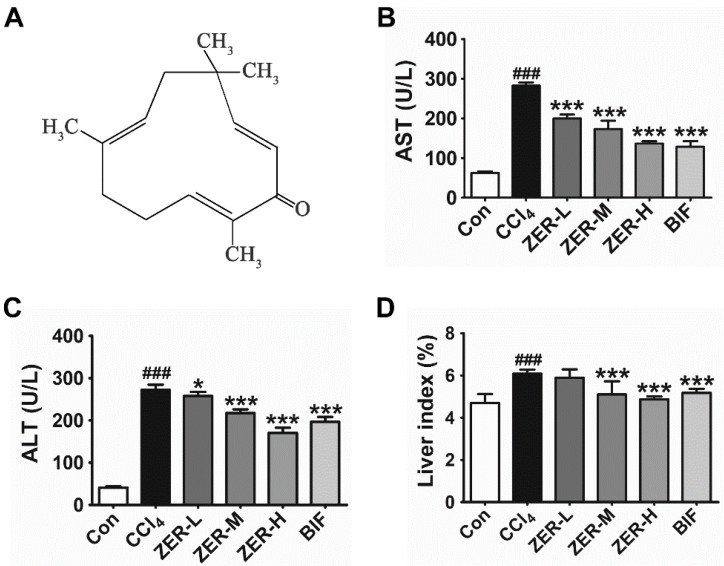
The structure of ZER (**A**) and the inhibitory effects of ZER on serum aspartate aminotransferase AST (**B**), alanine aminotransferase ALT (**C**), and liver index (**D**) on CCl_4_-induced acute liver injury (ALI) in mice. ZER-L, low dose group; ZER-M, medium dose group; ZER-L, high dose group. Values represent the mean ± SD (*n* = 10). ^###^ *p* < 0.001 compared with the control group; * *p* < 0.05 and *** *p* < 0.001 compared with the CCl_4_-treated group.

The structure of ZER is shown in Figure 1A. Bifendate (BIF) was used as a positive control, which is widely prescribed to treat hepatic injury. The mice were pretreated with ZER for five consecutive days and then challenged with a one-time intraperitoneal injection. The protective effects of ZER were first assessed by serum biochemical parameters. As shown in Figure 1B and Figure 1C, the activities of serum AST and ALT were markedly elevated in the CCl_4_ injection group with respect to the control group, indicating that the model of ALI induced by CCl_4_ was successfully established. ZER pretreatment effectively lowered the increase of serum AST and ALT activities in a dose-dependent manner. The ALT activity in the 20 μmol/kg ZER group was even lower than the BIF-positive control group. These results suggested that the CCl_4_-induced ALI mice model was successfully established and ZER harbors hepatoprotective properties against CCl_4_-induced ALI.

Liver disease usually accompanied the rise of liver index. In the CCl_4_-intoxicated animals, we also observed the increase of the liver index with respect to the normal group, which is shown in Figure 1D. Unsurprisingly, ZER pretreatment markedly decreased the liver index in a dose-dependent fashion, as demonstrated by the gradual decrease of the liver index with respect to that of the CC1_4_ group.

### 2.2. Histopathological Effect of ZER in Liver

**Figure 2:**
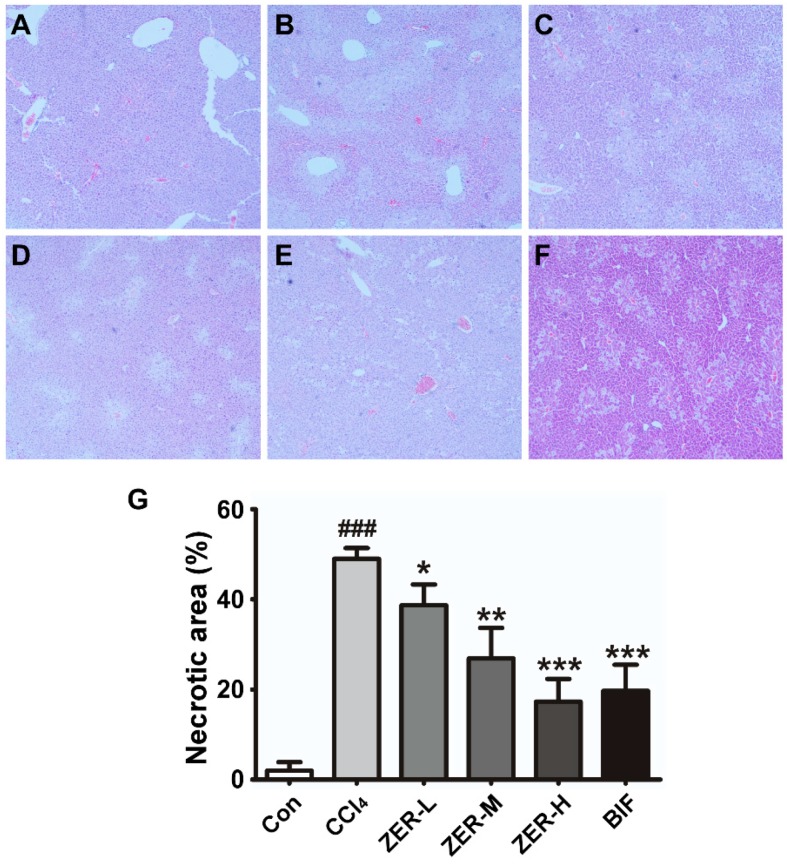
Protective effects of ZER on liver histopathology in CCl_4_-induced ALI mice. Liver tissues were stained with Hematoxylin and Eosin (H&E) staining (40×). (**A**), control group; (**B**), CCl_4_ group; (**C**), ZER-L group; (**D**), ZER-M group; (**E**), ZER-H group; (**F**), Bifendate (BIF) group. The pale pink areas are the necrotic areas. (**G**) Quantification of liver necrosis in histological sections (*n* = 10). Values represent the mean ± SD. ^###^ *p* < 0.001 compared with the control group; * *p* < 0.05, ** *p* < 0.01, and *** *p* < 0.001 compared with the CCl_4_-treated group.

To further assess the protective effects of ZER on CCl_4_-induced ALI, an examination of the liver histophathology was employed to evaluate the extent of the hepatic damage. After intoxication of the mice with CCl_4_, a moderate degree of hepatocyte necrosis was evident in the central lobular areas, which accounted for 49% (the model) of the liver section, indicating a successful mouse model of ALI (Figure 2B,G). However, ZER pretreatment significantly decreased the necrosis in a dose-dependent fashion (Figure 2C–G). The necrotic area decreased from 49% to 39.4% (ZER-L), 26.9% (ZER-M), or 19.7% (ZER-H) (Figure 2G). The histopathological results demonstrated that pretreatment with ZER effectively alleviated CCl_4_-induced centrolobular necrosis.

### 2.3. Effects of ZER on Oxidative Stress Parameters

**Figure 3:**
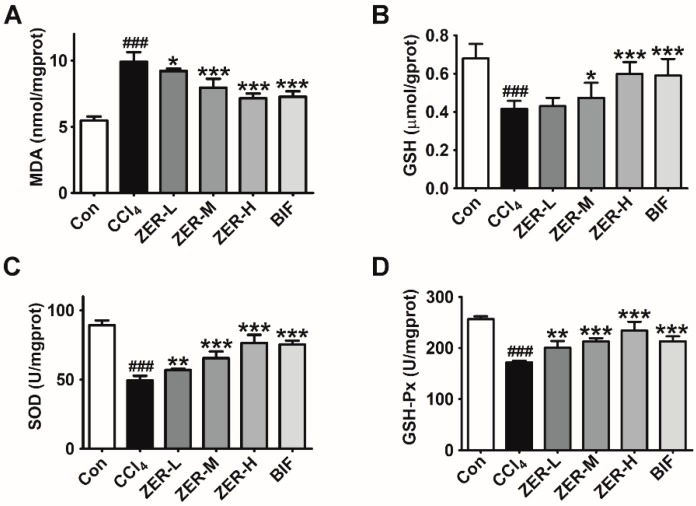
Effects of ZER on oxidative stress parameters (**A**) MDA, (**B**) GSH, (**C**) SOD, and (**D**) GSH-Px in liver tissue of CCl_4_-induced ALI mice. Values represent the mean ± SD (*n* = 10). ^###^ *p* < 0.001 compared with the control group; * *p* < 0.05, ** *p* < 0.01, and *** *p* < 0.001 compared with the CCl_4_-treated group.

It is well elucidated that oxidative stress is an important mechanism of action of hepatotoxicity induced by CCl_4_. CCl_4_ intoxication causes increasing generation of reactive oxygen species (ROS) in hepatocytes, which triggers GSH depletion and lipid peroxidation. MDA is the end product of lipid peroxidation and can be used as a marker for oxidative stress. ROS also results in a significant decrease in antioxidant enzyme activity, including SOD and GSH-Px. To understand the potential mechanisms of the observed hepatoprotective effects, oxidative stress parameters was evaluated by measuring MDA levels, GSH levels, and the activities of SOD and GSH-Px. As can be seen in Figure 3, MDA contents was remarkably increased, GSH levels were remarkably decreased, and SOD and GSH-Px activities were significantly decreased comparing to those in the control group after intoxication with CCl_4_. However, with ZER pretreatment, the generation of MDA in liver tissues was reduced, the depleted GSH levels were restored, and the decreased SOD and GSH-Px activities were increased, all in a dose-dependent manner. These results suggest that ZER exerts hepatoprotective effects by alleviating oxidative stress.

### 2.4. Effects of ZER on the Levels of TNF-α and IL-6 In Vivo

**Figure 4:**
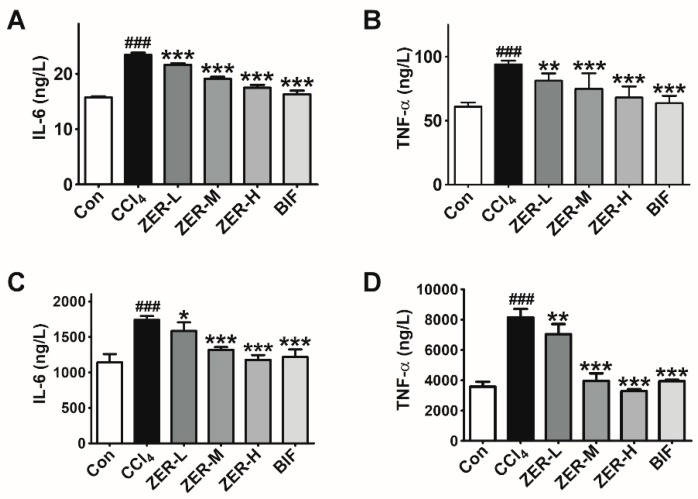
Effects of ZER on the levels of IL-6 ((**A**) serum and (**C**) liver tissue) and TNF-α ((**B**) serum and (**D**) liver tissue) in CCl_4_-induced ALI mice. Values represent the mean ± SD (*n* = 10). ^###^ *p* < 0.001 compared with the control group; * *p* < 0.05, ** *p* < 0.01, and *** *p* < 0.001 compared with the CCl_4_-treated group.

Besides oxidative stress, CCl_4_-induced ALI is also characterized by an inflammatory response due to pro-inflammatory cytokines such as TNF-α and IL-6 released by damaged hepatocytes, sinusoidal endothelial cells, and activated Kupffer cells. To investigate if ZER provides protection against CCl_4_-induced ALI via anti-inflammation, the change in levels of two main pro-inflammatory cytokines, TNF-α and IL-6, was examined. As can be seen in Figure 4, both serum and liver tissue TNF-α and IL-6 levels were elevated in the CCl_4_-intoxication group than in the control group, as determined by ELISA. However, pretreatment with ZER inhibited TNF-α and IL-6 production with respect to the CCl_4_-intoxication group in a dose-dependent manner. The results clearly showed that ZER-pretreatment could inhibit the CCl_4_-intoxication-induced production of inflammatory cytokines TNF-α and IL-6. These results hint that the hepatoprotective effect of ZER is achieved by down-regulating the inflammatory response.

### 2.5. Effects of ZER on TLR4/NF-κB/COX-2 Signaling In Vivo

**Figure 5:**
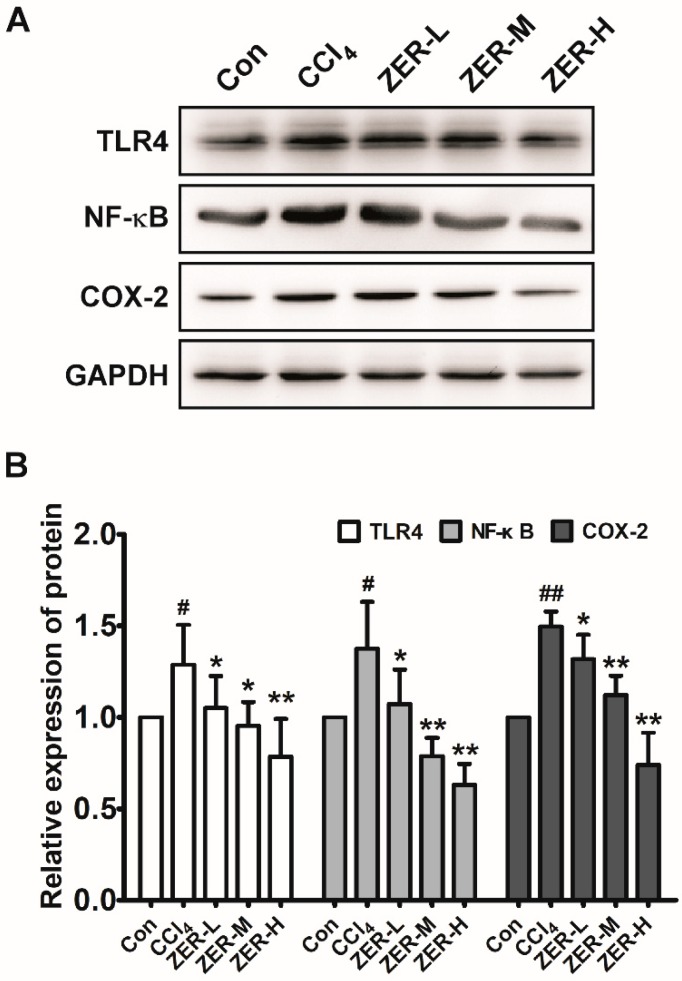
Effects of ZER on the protein levels of TLR4, NF-κB (p-p65), and COX-2 in liver tissue of CCl_4_-induced ALI mice. (**A**) Western blotting analysis of TLR4, NF-κB (p-p65), and COX-2; Glyceraldehyde-3-phosphate dehydrogenase (GAPDH) was used as the loading control. (**B**) Quantification of the relative protein levels of TLR4, NF-κB (p-p65), and COX-2. Values represent the mean ± SD (*n* = 3). ^#^ *p* < 0.05 and ^##^ *p* < 0.01 compared with the control group; * *p* < 0.05 and ** *p* < 0.01 compared with the CCl_4_-treated group.

We further explored the underlying anti-inflammatory mechanism of ZER against ALI induced by CCl_4_. As is well-known, the production of pro-inflammatory cytokines TNF-α and IL-6 are induced by activation of NF-κB signaling, so we first detected the change in the level of phosphorylated p65, which forms the most common heterodimer of NF-κB together with p50. As shown in Figure 5, compared with the control group, the protein level of p-p65 was increased in the liver tissue of the CCl_4_-challenged model group. The levels of p-p65 in the ZER-pretreated groups were decreased compared with that of the model group and the decrement was exhibited in a dose-dependent manner. We then detected the change in the levels of TLR4 and COX-2 proteins, which are the upstream and downstream molecules of the NF-κB pathway, respectively. The change in the levels of TLR4 and COX-2 were in accordance with that of p65, as can be seen in Figure 5. These results suggested that CCl_4_ intoxication may mediate the inflammatory response via TLR4/NF-κB/COX-2 signaling and ZER could inhibit inflammation by blocking the activation of the TLR4/NF-κB/COX-2 signaling pathway.

### 2.6. Effects of ZER on TLR4/NF-κB/COX-2 Signaling In Vitro

**Figure 6:**
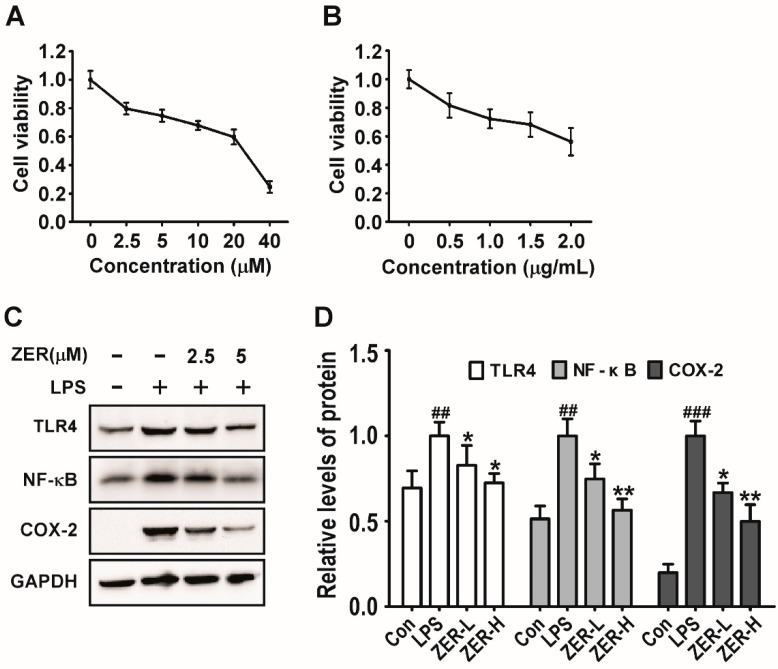
Effects of ZER on TLR4/NF-κB/COX-2 signaling in LPS-induced Raw264.7 cells. (**A**) The cytotoxicity of ZER was evaluated by MTT assay. (**B**) The cytotoxicity of LPS was evaluated by MTT assay. (**C**) Western blotting analysis of TLR4, NF-κB (p-p65), and COX-2; GAPDH was used as the loading control. (**D**) Quantification of the relative protein levels of TLR4, NF-κB (p-p65), and COX-2. Values represent the mean ± SD (*n* = 3). ^##^ *p* < 0.01 and ^###^ *p* < 0.001 compared with the control group; * *p* < 0.05 and ** *p* < 0.01 compared with the LPS treated group.

The expression analysis of the liver tissue samples suggested that ZER protects CCl_4_-induced ALI by ameliorating the inflammatory response through TLR4/NF-κB/COX-2 signaling. To further verify the involvement of this pathway in vitro, anLPS-induced Raw264.7 inflammatory cellular model was established and employed. We first assessed the potential inhibitory effects of ZER and LPS on cell viability against Raw264.7 cells at different concentrations. As shown in Figure 6A, the 75% viability of ZER inhibition of cell viability was observed at about 7.5 μmol/L and 50% at about 25 μmol/L. According to the 3-[4,5-dimethylthiazol-2-yl]-2,5 diphenyl tetrazolium bromide (MTT) results, we set two ZER treatment groups: 2.5 μmol/L as the low dose group and 5 μmol/L as the high dose group. As shown in Figure 6B, the cell viability of Raw264.7 cells treated with 0.5 μg/mL LPS for 36 h was about 80%. We further determined the activation situation of TLR4/NF-κB/COX-2 signaling upon ZER pretreatment in LPS-stimulated Raw264.7 cells. The results showed that LPS stimulation significantly activated the TLR4/NF-κB/COX-2 signaling, as evidenced by the up-regulated protein levels of TLR4, p-p65, and COX-2. Unsurprisingly, after treatment with different concentrations of ZER, the protein levels of TLR4, p-p65, and COX-2 were markedly decreased, as shown in Figure 6C,D. These results clearly confirmed that ZER could inhibit inflammation by blocking the activation of the TLR4/NF-κB/COX-2 signaling pathway.

## 3. Discussion

ALI, which is caused by a number of factors, such as toxic chemicals, drugs, viruses, and bacteria, seriously threatens human health [1,4]. Recently, great attention and effort have been devoted to discovering phytochemicals with preventive or therapeutic effects against ALI [6,7,11,12,16,17,30]. In the current study, we found that ZER exhibits a hepatoprotective activity against CCl_4_-induced ALI by reducing the level of oxidative stress and inhibiting the inflammatory response. Further results revealed that the anti-inflammation mechanism for ZER against CCl_4_-induced ALI involved inactivation of the TLR4/NF-κB/COX-2 signaling pathway. Together, these findings explain the pharmacological mechanism of ZER acting on CCl_4_-induced ALI and provided an experimental and theoretical basis for the application of ZER in the clinical treatment of ALI.

The decreased serum levels of ALT and AST upon ZER pretreatment imply the hepatoprotective effect against CCl_4_-induced ALI and pathological analysis further confirmed this beneficial effect by effectively decreasing the area of centrolobular necrosis. When trying to elucidate the underlying mechanism, two pathogenic events were taken into consideration, oxidative stress and inflammation, since these two events during ALI normally serve as the mechanisms for liver damage [4,32]. Meanwhile, ZER is reported to possess many pharmacological properties, including antioxidant and anti-inflammatory properties [33]. Therefore, we hypothesized that ZER would exert hepatoprotective activity against ALI by alleviating oxidative stress and the inflammatory response. In light of the assessment of oxidative stress parameters in the liver tissues, including SOD, GSH-Px, GSH, and MDA, we confirmed that ZER exerts hepatoprotective effects against CCl_4_-induced ALI through reducing oxidative stress by recovering the activities of antioxidant enzymes, restoring the GSH reservoir, and reducing lipid peroxidation specifically. Afterward, the evaluation of the change in serum and hepatic levels of pro-inflammatory cytokines IL-6 and TNF-α upon ZER pretreatment confirmed the involvement of anti-inflammation for ZER against ALI, which is consistent with Kim et al.’s report [30]. Based on this observation, we tried to seek the signaling pathways responsible for the protection of ZER against ALI. Protein expression analysis of liver tissue samples hinted at the TLR4/NF-κB/COX-2 signaling pathway. Further in vitro studies in Raw264.7 cells verified the involvement of this pathway, as evidenced by the reduced release amounts of IL-6 and TNF-α and down-regulated protein levels of TLR4, NF-κB, and COX-2, which were originally elevated upon LPS stimulation. Thus, the anti-inflammation activity for ZER against CCl_4_-induced ALI may occur through the TLR4/NF-κB/COX-2 signaling pathway.

Antioxidant enzymes, such as SOD and GSH-Px plus GSH, which are scavengers of ROS in the liver, are the first line of defense against oxidative stress-induced tissue injury [34]. Several previous studies have shown that ZER can inhibit the generation of ROS [35,36,37,38]. For instance, ZER was found to provide protection to pancreatic β cells from high glucose-induced cytotoxicity through directly interfering with ROS production [39]. Our data also suggested this inhibition of ROS generation. However, contrary observations have been reported. For example, treatment with ZER has been found to significantly increase reactive oxygen species levels in colorectal cancer cells and melanoma cells [40,41]. These discrepancies may arise from distinct disease contexts and different cellular processes involved in the studies; the observations of ZER in favor of ROS generation were implicated in apoptosis under the cancer context [40,41,42,43,44], whereas the observations of ZER against ROS generation were mostly related to inflammation and inflammatory-associated diseases, such as hepatic disease, osteoarthritis, and diabetes and diabetic complications [22,24,39,45].

An excessive inflammatory response can aggravate injured hepatic tissue and hinder repair processes [13,32]. Previous studies have reported the hepatoprotective activity of ZER against ALI via anti-inflammation [30,31]. However, our results further suggested that ZER might inhibit CCl_4_-induced inflammation through TLR4/NF-κB/COX-2 signaling. There are studies in the literature that support our observations. An in vitro study conducted in human leukemic cell line THP-1 cell-derived macrophages activated by LPS showed that ZER inhibits the secretion of pro-inflammatory cytokines TNF-α and IL-6, the induction of NF-κB and COX-2, and also decreases the elevated mRNA levels of TLR2 and TLR4 [46]. Lee and Haque reported the inhibition of NF-κB p65 phosphorylation and pro-inflammatory cytokines release by ZER, respectively [27,47]. It is well established that hepatic macrophages, including activated Kupffer cells, hold central functions in initiating, perpetuating, and even restricting inflammation in the liver [10,13]. According to our results both in vivo and in vitro, it is likely that ZER targets hepatic macrophages to inhibit unregulated inflammatory responses, thus preventing further hepatic injury and promoting hepatic tissue repair. However, this hypothesis needs to be tested.

Hepatocellular injury leads to cell death, including both necrosis and apoptosis [48,49,50]. However, uncontrolled necrosis and apoptosis can provoke further hepatic damage [30,51]. Our results show that ZER pretreatment effectively decreases the necrotic area of the injured liver by inhibiting the inflammatory response through the TLR4/NF-κB/COX-2 signaling pathway. Meanwhile, Kim et al. reported that ZER exerts a hepatoprotective activity by inhibiting apoptosis through inhibiting the translocation of activated hepatic JNK to mitochondria, thus ameliorating CCl_4_-intoxication-induced oxidative stress. Hence, ZER provides protection against CCl_4_-induced ALI via alleviating both necrosis and apoptosis.

The present study revealed that ZER, which is a monocyclic sesquiterpene [30], provided protection against ALI through ameliorating oxidative stress and inflammation. Meanwhile, several previous studies showed that sesquiterpene or sesquiterpene lactones exhibit hepatoproctive activities toward different types of hepatic disease [8,52,53,54]. Another two studies showed that a mixture with 21 kinds of sesquiterpenoids extracted from the root of *Panax Ginseng* exhibited strong hepatoproctive effects, which were also mediated by reducing oxidative stress and inhibiting the inflammation response [7]. In fact, more and more sesquiterpenoids, including sesquiterpenes and sesquiterpene lactones, have been reported as having either anti-oxidation or anti-inflammation activities, or both [7,22,55,56]. Given that oxidative stress and inflammation are usually present simultaneously and intertwined to aggravate already-existing liver tissue damage [4,13] and the clinical consideration and therapeutic strategy for liver diseases predominantly focuses on anti-oxidation and anti-inflammation [1,14], we speculate that natural products of sesquiterpenoids extracted from plants may be a promising reservoir to screen effective therapeutic drugs for hepatic disease with high efficacy and low toxicity.

Collectively, our work revealed a new mechanism of ZER against ALI, which is through ameliorating oxidative stress. Furthermore, unlike the previous study, we unraveled the involvement of inactivation of the TLR4/NF-κB/COX-2 signaling pathway in the modulation of the inflammatory response upon ZER pretreatment. We also acknowledge that the validation data about the involvement of TLR4/NF-κB/COX-2 signaling was only conducted in vitro and so may not be convincing. Employment of an NF-κB inhibitor in vivo is a better way to confirm the correlation. Last but not least, a better understanding of the mechanism underlying the hepatoprotective activity of ZER against ALI is very necessary so that successful translation of ZER to clinical applications can be developed rapidly for liver disease.

In conclusion, our study raises the possibility that ZER could be used as a potential therapeutic drug for hepatic disease.

## 4. Materials and Methods

### 4.1. Chemicals and Reagents

Zerumbone (ZER) was purchased from Sigma Aldrich (No.Z3902, St. Louis, MO, USA), dissolved in DMSO (Dimethyl sulfoxide). Bifendate (BIF) was purchased from Beijing Yuehe Pharmaceutical Factory (H11020980, Beijing, China). LPS was purchased from Beyotime Biotechnology (No.S1732, Shanghai, China). RPMI 1640 Medium was purchased from Hyclone (Logan, UT, USA). Fetal bovine serum (FBS) was purchased from Gibco (Gaithersburg, MA, USA). The assay kits for ALT, AST, SOD, GSH-Px, GSH, and MDA were purchased from Nanjing Jiancheng Bioengineering Institute (Nanjing, China). Bicinchoninic acid (BCA) assay kit and MTT were purchased from Solarbio (Beijing, China). Mouse IL-6 and TNF-α ELISA kits was purchased from ShangHai Haling Biological Technology Co., Ltd. (ShangHai, China). Rabbit anti-TLR4 antibody (#14358, 1:1000), rabbit anti-COX-2 antibody (#4842, 1:1000), rabbit anti-p-NF-κB p65 (Ser536) antibody (#3033, 1:1000), mouse anti-β-Actin antibody (#3700, 1:2000) were purchased from Cell Signaling Technology (Danvers, MA, USA). All other chemicals and reagents were purchased from local firms.

### 4.2. Animals

Specific pathogen-free (SPF) male ICR mice (SCXK20140007), weighting 20 ± 2g, 7~8 weeks, were purchased from Jinan Pengyue Experimental Animal Breeding Co., Ltd. (Jinan, Shandong, China). The mice were kept in the Laboratory Animal Centre of the First Affiliated Hospital of Henan University of Science and Technology. The feeding conditions for the mice were provided with a constant temperature of 22 ± 2 °C and a relative humidity of 55 ± 5% with a 12 h/12 h light–dark cycle. During the experiment, the mice were given a standard diet with clean mineral water and pellet feed. All experiments were conducted in accordance with the Guide for the Care and Use of Laboratory Animals and the animal experiment protocols were approved by the Institutional Ethical Committee of Henan University of Science and Technology.

### 4.3. Experimental Design

After a five-day acclimation based on body weight, sixty ICR mice were equally divided into six groups of 10 animals each, as follows: The control (Con) group, the CCl_4_ model group, the ZER-L (1.25 μmol/kg of body weight (BW), dissolved in DMSO) group, the ZER-M (5 μmol/Kg) group, the ZER-H (20 μmol/Kg) group, and the positive control group treated with BIF (350 μmol/Kg/day). The final concentration (*v/v*) of DMSO in the in vivo experiment was 1%. During the experiment, the mice were given a standard diet. All mice were intraperitoneally injected with corresponding drugs for 5 consecutive days: The control group and the CCl_4_ model group injected with DMSO, three ZER groups with ZER with the corresponding concentrations, and the positive control group with BIF. Two hours after the last administration, all the animals from each group received a one-time intraperitoneal injection of CCl_4_ (0.01 mL/g body weight, 0.2% in soybean oil) to establish the ALI model, except for the control group, which were received the same volume of soybean oil. Twelve hours fasting followed with the CCl_4_ challenge.

The mice were sacrificed under anesthesia (6% chloral hydrate, 10 mL/kg) 12 h after the CCl_4_ challenge. Blood and liver tissue were collected for experiments. The serum was obtained by centrifuging (3000 rpm, 4 °C, 15 min) and then immediately subjected to the following biochemical analysis. For the liver, a whole lobe without any mechanical damage was fixed in 10% formalin for histopathological analysis, while the rest of the liver tissue samples were stored in liquid nitrogen for further experiments.

### 4.4. Detections of Biochemistry Indexes

The activities of ALT and AST in serum and the activities or levels of SOD, GSH-Px, GSH, and MDA in liver homogenates were determined by commercial kits according to the manufacturer’s instructions. The 10% liver homogenates were prepared as follows: The liver tissues were homogenized in nine-fold (*w/v*) cold saline on ice and then centrifuged (12,000 rpm, 30 min, 4 °C), supernatants were collected for detection.

### 4.5. Histopathological Analysis

The liver lobes fixed in 10% formalin were embedded in paraffin and processed into 4 μm thick sections, which were subjected to H&E staining. All sections were examined and pictured under an optical microscope. Areas of necrosis were quantified using ImageJ v1.8.0 software (National Institutes of Health, Bethesda, MD, USA).

### 4.6. Detections of Inflammatory Cytokines IL-6 and TNF-α

The levels of IL-6 and TNF-α both in serum and in liver tissue were analyzed by ELISA kits according to the manufacturer’s instructions. The serum was prepared as in Section 4.3 and 10% liver homogenates were prepared as in Section 4.4.

### 4.7. Cell Culture and LPS Treatment

The Raw264.7 cell line was a generous gift from Dr Ruifang Li, who also works in Medical School of Henan University of Science and Technology. The Raw264.7 cell line was cultured in Roswell Park Memorial Institute (RPMI) 1640 medium supplemented with 10% (*v/v*) FBS and antibiotics (100 U/mL penicillin G and streptomycin sulfate) in an incubator at 37 °C with 5% CO_2_ and saturated humidity. ZER was dissolved in DMSO to a concentration of 10 mM for the in vitro experiment. The final concentration (*v/v*) of DMSO in the in vivo experiment was 0.05%. For LPS stimulation, the dosage and timing for activating the Raw264.7 cells was 0.5 μg/mL LPS for 36 h. The cells in the exponential growth phase were harvested and seeded into 6-well plates at 6 × 10^5^ cells per well, then stimulated by LPS (0.5 μg/mL) for 12 h when the cells were of 80% confluence. ZER was then added for another 24 h. The cells were lysed and subjected to Western blotting analysis.

### 4.8. Cytotoxicity Assessment of ZER and LPS by MTT Assay

The cytotoxicity of ZER and LPS toward Raw264.7 cells were evaluated by MMT assays. The procedure was carried out as previously described with little modification [57]. Raw264.7 cells were seeded into 96-well plates with 1 × 10^4^ cells in 100 μL medium per well overnight. Then, the cells were treated with different concentrations of ZER (0, 2.5, 5, 10, 20, and 40 μmol/L) for 24 h or different concentrations of LPS (0, 0.5, 1.0, 1.5, and 2.0 μg/mL) for 36 h. The control group was treated with DMSO or ddH_2_O. Subsequently, 15 μL of MTT (5 mg/mL, Sigma, St. Louis, MO, USA) was added to each well and 4 h later, the medium was discarded and the purple formazan crystals were solubilized in 150 μL DMSO per well. Absorbance was detected at 490 nm on Microplate Readers (Bio-Tek, USA). The viability rate was calculated as follows: Viability rate (%) = (A _model_/A _control_) × 100%.

### 4.9. Western Blotting Analysis

This procedure was described in our previous study. The total proteins of liver tissue or Raw264.7 cells were extracted by Radio Immunoprecipitation Assay (RIPA) lysis solution (Solarbio, Beijing, China) with phenylmethanesulfonyl fluoride (PMSF) on ice following the user guide. BCA protein content determination was used for quantification. The same amount of protein (40 μg) was subjected to Western blotting analysis as previously described [53]. The signals were detected by the Western Bright^TM^ electrochemiluminescence (ECL)kit (Advansta, Menlo Park, CA, USA), then acquired and analyzed by Image Lab Software (Bio-RadLaboratories, Hercules, CA, USA).

### 4.10. Statistical Analysis

All experimental data were expressed as mean ± SD (standard deviation). Statistical significance was determined by SPSS 16.0 software (IBM,.Chicago, IL, USA), using one-way analysis of variance (ANOVA) with LSD Fisher post hoc test. *p* < 0.05 was considered as significant.

## Abbreviations

CCl_4_	Carbon tetrachloride
ALI	Acute liver injury
ZER	Zerumbone
AST	Aspartate aminotransferase
ALT	Alanine aminotransferase
SOD	Superoxide dismutase
GSH-Px	Glutathione peroxidase
GSH	Glutathione
MDA	Malondialdehyde
IL-6	Interleukin-6
TNF-α	Tumor necrosis factor alpha
TLR4	Toll-like Receptor 4
NF-κB	Nuclear factor -κB
COX-2	Cyclooxygenase-2
TLRs	Toll-like receptors
HMGB1	High mobility group box-1 protein
DAMPs	Damage associated molecular patterns
BIF	Bifendate
LPS	Lipopolysaccharide
ROS	Reactive oxygen species
FBS	Fetal bovine serum
H&E	Hematoxylin and Eosin
